# Editorial: Brain Metastases (Secondary Brain Tumor)

**DOI:** 10.3390/cancers17213440

**Published:** 2025-10-27

**Authors:** Dai Shida

**Affiliations:** Division of Frontier Surgery, The Institute of Medical Science, The University of Tokyo, 4-6-1 Shirokanedai, Minato-ku, Tokyo 108-8639, Japan; dshida@g.ecc.u-tokyo.ac.jp; Tel.: +81-3-3443-8111

## 1. Editorial: Brain Metastases (Secondary Brain Tumor)

Brain metastases, the most common intracranial neoplasms in adults, remain a major cause of cancer-related mortality and morbidity [1]. Neurological deterioration due to tumor invasion markedly impairs patients’ quality of life. The incidence of brain metastases is increasing, reflecting improved MRI detection and prolonged survival from advances in systemic therapy [2]. As our biological understanding and therapeutic armamentarium expand, management strategies have shifted from palliative to precision-integrated care [3]. This Special Issue highlights current advances and future directions in the multidisciplinary treatment of brain metastases originating from diverse primaries, including lung, renal, breast, gynecologic, and colorectal cancers.

## 2. A Brief Overview of Recent Developments in the Field

Historically, whole-brain radiotherapy was the standard treatment for multiple brain metastases, providing intracranial control at the cost of cognitive decline. Over the past two decades, high-resolution MRI and stereotactic radiosurgery have transformed local therapy, enabling precise, minimally invasive management. The landmark JLGK0901 study demonstrated that stereotactic radiosurgery alone achieves comparable outcomes in patients with up to ten brain metastases, establishing that the total tumor volume, rather than lesion count, determines eligibility [4]. Similarly, the JCOG0504 phase III trial confirmed the noninferiority of postoperative stereotactic radiosurgery to whole-brain radiotherapy in overall survival (median 15.6 months each; HR = 1.05) while reducing neurotoxicity and preserving cognition [5]. These pivotal studies redefined global standards, making stereotactic radiosurgery with regular MRI follow-up the preferred postoperative approach for patients with ≤4 lesions [6].

Concurrently, the evolution of systemic therapy has profoundly influenced intracranial disease management. Tyrosine kinase inhibitors (TKIs) and immune checkpoint inhibitors (ICIs) have demonstrated significant activity against brain lesions in cancers such as non-small-cell lung cancer, melanoma, and HER2-positive breast cancer, particularly in patients with EGFR, ALK, ROS1, or BRAF mutations [7]. Integrating these systemic agents with focal therapies has improved survival and intracranial control, promoting the transition toward individualized, multimodality care [8].

## 3. The Gap in Knowledge

Despite these advances, key gaps persist. Current evidence remains limited regarding the optimal sequencing of local and systemic therapies, the mechanisms underlying therapeutic resistance within the central nervous system, and strategies to overcome the blood–brain barrier. Neurocognitive outcomes also require more refined assessment, as conventional tools, such as the Mini-Mental State Examination (MMSE), may fail to detect subtle yet clinically significant deficits. Moreover, biological studies reveal that brain metastases harbor unique molecular and microenvironmental adaptations, underscoring the need for deeper genomic and translational research to identify central nerve system-specific therapeutic targets.

Collectively, these developments have reshaped the clinical paradigm—from uniform palliative treatment to biologically informed precision strategies aimed at maximizing tumor control while preserving neurological function and quality of life.

## 4. How This Special Issue Addressed Those Gaps

The *Cancers* Special Issue “Brain Metastases (Secondary Brain Tumor)” brought together ten original and review articles that collectively bridged the translational and clinical divide in brain metastases research. These works addressed critical gaps—from the molecular evolution of intracranial disease to the clinical integration of systemic therapy, surgery, and radiosurgery.

At the molecular level, Váraljai R. et al. made an outstanding contribution by demonstrating that intracranial melanoma metastases harbor distinct driver gene alterations—including ARID1A, ARID2, SMARCA4, and BAP1—that differ from those in extracranial lesions, supporting a branched evolutionary model of metastatic progression. These findings shed light on brain-specific genomic adaptations underlying central nervous system colonization and hold promise for the development of novel biomarkers and targeted therapeutic strategies.

Clinically, several studies highlighted the synergy between local and systemic therapies. Skribek M. et al. reported real-world evidence that ICIs achieve durable intracranial control even in neurologically symptomatic NSCLC, while Cho A. et al. showed that concurrent administration of ICIs or TKIs with Gamma Knife radiosurgery significantly prolongs survival without increasing radiation necrosis or hemorrhagic risk. Together, these studies establish radiosurgery as a powerful partner to systemic immunotherapy, enabling a transition from palliative intent to integrative disease management.

Surgical and perioperative insights were provided by Schneider M. et al., who identified comorbidity burden and lesion multiplicity as independent predictors of postoperative complications, and by Lange N. et al., who revealed perioperative metabolic factors—such as elevated lactate and glucose—as prognostic indicators for infarction and reduced survival. These findings underscore the need for meticulous perioperative optimization and metabolic monitoring.

Complementing these original works, several comprehensive reviews broadened disease-specific understanding of brain metastases:

- Watase C. et al. provided an in-depth overview of brain metastases from breast cancer, addressing pathophysiology, diagnostic advances, local treatments (surgery, stereotactic radiosurgery, whole-brain radiotherapy), and emerging subtype-specific systemic therapies capable of crossing the blood–brain barrier.
- Kato M. K. et al. [9] summarized brain metastases from uterine cervical and endometrial cancers, for which no targeted or nanomedicine-based therapies currently exist, emphasizing the potential benefit of surgical resection and stereotactic modalities even in this rare context.
- Matsui Y. et al. [10] reviewed brain metastases from renal cell carcinoma, highlighting how ICIs and molecular-targeted agents have reshaped the therapeutic landscape and advocating their integration with stereotactic radiosurgery.
- Borella F. et al. analyzed brain metastases arising from ovarian cancer and identified BRCA mutations and low androgen receptor expression in the primary tumor as emerging risk and prognostic factors, suggesting that these patients could benefit from tailored surveillance strategies.
- Finally, Müller S. et al. investigated brain metastases from colorectal cancer and found that the vast majority of patients (up to 96%) are asymptomatic, often leading to delayed diagnosis, thereby supporting the implementation of screening protocols for high-risk populations.

Collectively, these contributions underscore the strength of an integrative perspective—bridging molecular biology, clinical innovation, and tumor-specific insight. By encompassing both common and rare primary tumors, this Special Issue not only deepens our scientific understanding but also expands the clinical vision of brain metastasis management toward truly personalized, multidisciplinary care.

## 5. Future Research Directions

Despite remarkable progress, brain metastases remain a formidable challenge characterized by biological heterogeneity, therapeutic resistance, and limited survival. Building upon the insights from this Special Issue, several priorities should guide future research.

First, comprehensive multi-omics analyses of paired primary, extracranial, and intracranial tumors are essential to elucidate central nervous system tropism and uncover actionable biomarkers for brain-penetrant therapeutics. Second, the demonstrated synergy between stereo-tactic radiosurgery and systemic therapies, such as ICIs or TKIs, warrants prospective trials to define optimal sequencing, timing, and patient selection based on molecular and immunologic profiles. Third, refining surgical and perioperative strategies—through risk modeling, intraoperative imaging, and enhanced recovery protocols—may improve outcomes and reduce morbidity

## 6. Conclusions

This Special Issue has significantly expanded the knowledge base surrounding brain metastases—uniting molecular biology, clinical innovation, and multidisciplinary management. Yet, the field stands at a pivotal moment: to move from descriptive understanding to predictive, preventive, and personalized strategies. Future progress will depend on integrative science that combines genomic discovery, precision therapeutics, and patient-centered outcome research. Through sustained collaboration between laboratory scientists, neurosurgeons, radiation oncologists, and medical oncologists, the ultimate goal—prolonged survival with preserved neurological function—is within reach for patients facing this formidable manifestation of cancer.

## References

[1] Barnholtz-Sloan J.S., Sloan A.E., Davis F.G., Vigneau F.D., Lai P., Sawaya R.E. (2004). Incidence proportions of brain metastases in patients diagnosed (1973 to 2001) in the Metropolitan Detroit Cancer Surveillance System. J. Clin. Oncol..

[2] Imaizumi J., Shida D., Narita Y., Miyakita Y., Tanabe T., Takashima A., Boku N., Igaki H., Itami J., Kanemitsu Y. (2019). Prognostic factors of brain metastases from colorectal cancer. BMC Cancer.

[3] Imaizumi J., Shida D., Boku N., Igaki H., Itami J., Miyakita Y., Narita Y., Takashima A., Kanemitsu Y. (2023). Prognostic factors associated with the transition in treatment methods for brain metastases from colorectal cancer. Int. J. Clin. Oncol..

[4] Yamamoto M., Serizawa T., Shuto T., Akabane A., Higuchi Y., Kawagishi J., Yamanaka K., Sato Y., Jokura H., Yomo S. (2014). Stereotactic radiosurgery for patients with multiple brain metastases (JLGK0901): A multi-institutional prospective observational study. Lancet Oncol..

[5] Kayama T., Sato S., Sakurada K., Mizusawa J., Nishikawa R., Narita Y., Sumi M., Miyakita Y., Kumabe T., Sonoda Y. (2018). Effects of Surgery with Salvage Stereotactic Radiosurgery Versus Surgery with Whole-Brain Radiation Therapy in Patients with One to Four Brain Metastases (JCOG0504): A Phase III, Noninferiority, Randomized Controlled Trial. J. Clin. Oncol..

[6] Narita Y., Sato S., Kayama T. (2022). Review of the diagnosis and treatment of brain metastases. Jpn. J. Clin. Oncol..

[7] Vogelbaum M.A., Brown P.D., Messersmith H., Brastianos P.K., Burri S., Cahill D., Dunn I.F., Gaspar L.E., Gatson N.T.N., Gondi V. (2022). Treatment for Brain Metastases: ASCO-SNO-ASTRO Guideline. J. Clin. Oncol..

[8] Suh J.H., Kotecha R., Chao S.T., Ahluwalia M.S., Sahgal A., Chang E.L. (2020). Current approaches to the management of brain metastases. Nat. Rev. Clin. Oncol..

[9] Kato M.K., Tanase Y., Uno M., Ishikawa M., Kato T. (2021). Brain Metastases from Uterine Cervical and Endometrial Cancer. Cancers.

[10] Matsui Y. (2020). Current Multimodality Treatments Against Brain Metastases from Renal Cell Carcinoma. Cancers.

